# Introduction of Exogenous Glycolate Catabolic Pathway Can Strongly Enhances Photosynthesis and Biomass Yield of Cucumber Grown in a Low-CO_2_ Environment

**DOI:** 10.3389/fpls.2019.00702

**Published:** 2019-05-29

**Authors:** Zhi-feng Chen, Xiu-ping Kang, Hong-mei Nie, Shao-wen Zheng, Tian-li Zhang, Dan Zhou, Guo-ming Xing, Sheng Sun

**Affiliations:** ^1^College of Horticulture, Shanxi Agricultural University, Jinzhong, China; ^2^Collaborative Innovation Center for improving the Quality and Efficiency of Greenhouse Vegetable in Shanxi Province, Taigu County, China

**Keywords:** *Cucumber*, glycolate catabolic pathway, low-CO_2_ treatment, multigene co-overexpression, high-photosynthetic-efficiency

## Abstract

Carbon dioxide (CO_2_) is very important for photosynthesis of green plants. CO_2_ concentration in the atmosphere is relatively stable, but it drops sharply after sunrise due to the tightness of the greenhouse and the absorption of CO_2_ by vegetable crops. Vegetables in greenhouses are chronically CO_2_ starved. To investigate the feasibility of using genetic engineering to improve the photosynthesis and yield of greenhouse cucumber in a low CO_2_ environment, five genes encoding glyoxylate carboligase (GCL), tartronic semialdehyde reductase (TSR), and glycolate dehydrogenase (GlcDH) in the glycolate catabolic pathway of *Escherichia coli* were partially or completely introduced into cucumber chloroplast. Both partial pathway by introducing GlcDH and full pathway expressing lines exhibited higher photosynthetic efficiency and biomass yield than wild-type (WT) controls in low CO_2_ environments. Expression of partial pathway by introducing GlcDH increased net photosynthesis by 14.9% and biomass yield by 44.9%, whereas the expression of the full pathway increased seed yield by 33.4% and biomass yield by 59.0%. Photosynthesis, fluorescence parameters, and enzymatic measurements confirmed that the introduction of glycolate catabolic pathway increased the activity of photosynthetic carbon assimilation-related enzymes and reduced the activity of photorespiration-related enzymes in cucumber, thereby promoting the operation of Calvin cycle and resulting in higher net photosynthetic rate even in low CO_2_ environments. This increase shows an improvement in the efficiency of the operation of the photosynthetic loop. However, the utilization of cucumber of low concentration CO_2_ was not alleviated. This study demonstrated the feasibility of introducing the pathway of exogenous glycolate catabolic pathway to improve the photosynthetic and bio-yield of cucumber in a low CO_2_ environment. These findings are of great significance for high photosynthetic efficiency breeding of greenhouse cucumber.

## Introduction

Applying solar energy-saving to greenhouses is the main strategy for counter-season production of vegetables in northern areas in north China in winter and spring. The greenhouses are often not ventilated due to the low external temperature outside (Kläring et al., 2007). Thus, the carbon dioxide (CO_2_) in greenhouses is consumed by vegetables after sunrise and rapidly decreases to a very low level. Long-term growth of greenhouse cucumber in low-concentration CO_2_ environment is characterized by low photosynthetic efficiency with low efficiency to utilize the light and heat resources (Slack and Hand, 1985). Most vegetables are C3 plants. RuBisCO is a key enzyme in photosynthesis that determines the rate of carbon assimilation in C3 plants (Raines, 2011). RuBisCO activity is affected by CO_2_ concentration. When the concentration of CO_2_ is high, RuBisCO catalyzes the carboxylation of RuBP and performs CO_2_ fixation. When the concentration of CO_2_ is low, RuBisCO catalyzes the oxygenation of RuBP to produce toxic glycolic acid, which is metabolized through photorespiration (Fichtner et al., 1993). The entire process of photorespiration is accompanied by the consumption of energy and carbon, thereby affecting the synthesis of sugars and the increase of biomass yield. According to the report, carbon consumed by photorespiration can account for 30–50% of the total carbon sequestration of photosynthesis in C3 plants (Farquhar et al., 1980). It is important to determine how to improve the utilization rate of low-concentration CO_2_ in vegetable crops in a low CO_2_ greenhouse environment and how to improve crop photosynthetic efficiency to promote sustainable, high, and stable yield of greenhouse vegetables. Improving vegetable yield and quality and effectively utilizing light and heat resources in greenhouse are important.

Cucumber is one of the world’s four main economic vegetables, and it plays an important role in world vegetable production. The CO_2_ compensation point of cucumber single leaf is 50–70 μmol⋅mol^-1^ (Zhang et al., 1997). Due to the large leaf area coefficient and the strong photosynthesis of cucumber, it could be affected by the CO_2_ concentration. If the greenhouse vent is completely open, the greenhouse CO_2_ concentration is 10% lower inside than outside. If the greenhouse vent is completely closed, the CO_2_ concentration will drop to 50–100 μmol⋅mol^-1^ (Slack and Hand, 1984). Cucumber can grow in a low CO_2_ environment for a long time and cannot effectively utilize the light and heat resources of greenhouse, resulting in low photosynthetic efficiency and reduced yield and quality.

*Escherichia coli* (*E. coli*) can use the glycolate as the sole carbon source, metabolize glycolate and release CO_2_ (Lord, 1972; Pellicer et al., 1996). According to the characteristics of *E. coli*, researchers introduced the glycolate catabolic pathway into *Arabidopsis thaliana* (Kebeish et al., 2007), *Camelina sativa* (Dalal et al., 2015), and *Solanum tuberosum* (Nölke et al., 2014) by expressing the *E. coli* glycolate metabolism-related enzyme genes. These authors proposed that the pathway could enhance the photosynthesis and growth of the corresponding plants due to a higher internal CO_2_ concentration around RuBisCO, thereby resulting in higher RuBisCO carboxylation activity (Kebeish et al., 2007; Nölke et al., 2014; Dalal et al., 2015).

**FIGURE 1:**
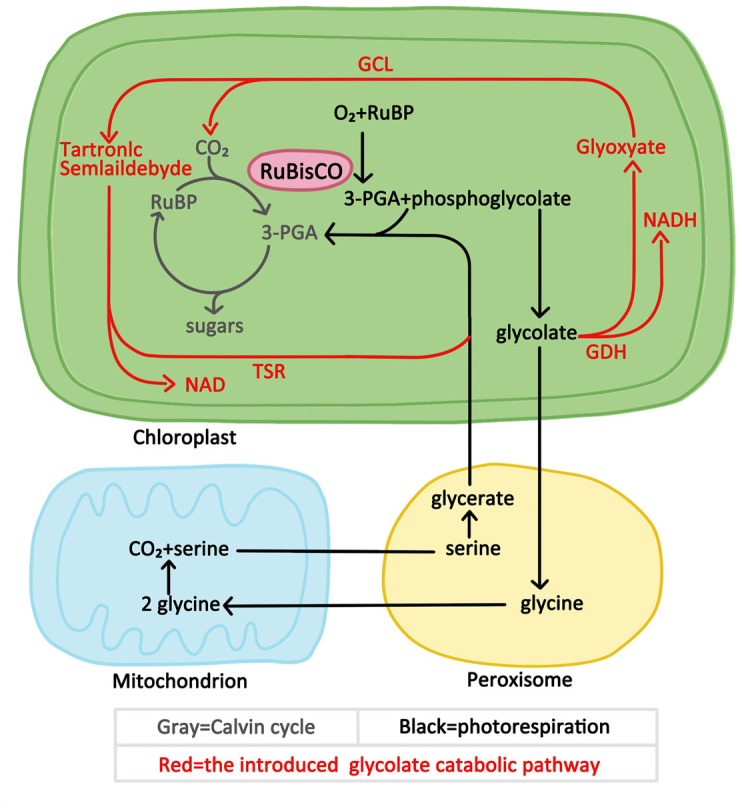
Superposition of *E. coli* glycolate catabolic pathway in plant photosynthetic carbon assimilation pathway.

This study aimed to verify whether the introduction of exogenous glycolate catabolic pathway had a positive effect on improving photosynthesis and growth of cucumber under low CO_2_ environmental conditions. We introduced either a partial or the full *E. coli* glycolate catabolic pathway into cucumber chloroplasts by expressing the genes encoding the *E. coli* glycolate catabolic enzymes (Figure 1). Several transgenic cucumber lines co-expressing glyoxylate carboligase gene (*GCL*) and tartronic semialdehyde reductase gene (*TSR*) or co-expression three subunit genes (*GlcD*, *GlcE*, and *GlcF*) of glycolate dehydrogenase (GlcDH) and plants co-expressing GlcD, GlcE, GlcF, GLC, and TSR were generated. We demonstrated that the introduction of *E. coli* glycolate catabolic pathway can improve the photosynthesis and increase the biomass yield of cucumber growing in a low-CO_2_ environment, which is of great significance for high photosynthetic efficiency breeding of greenhouse cucumber.

## Materials and Methods

### Plasmid, Bacteria and Plant Material

The cloning vector used in this study is PMD-18T (TransGen, China), and the plant expression vector used is pCamBIA 1305.1 (Wang et al., 2015b). The *E. coli* strain for gene cloning is DH5α (Takara, Japan). The *Agrobacterium tumefaciens* strain for genetic transformation of cucumber is C58C1. The plant material used for genetic transformation is cucumber inbred line 3407 (Wang et al., 2015a).

### Gene Synthesis and Vector Construction

The *GlcD*, *GlcE*, *GlcF*, *GCL*, and *TSR* genes encoding GlcDH, GCL, and TSR were from the *E. coli* K12 genome database (gi49175990) (Kebeish et al., 2007). The codons encoding the five genes were optimized by cucumber preference codon and synthesized. To facilitate subsequent Western blot detection, a flag protein tag was added to the 3′ end of each gene.

**FIGURE 2:**
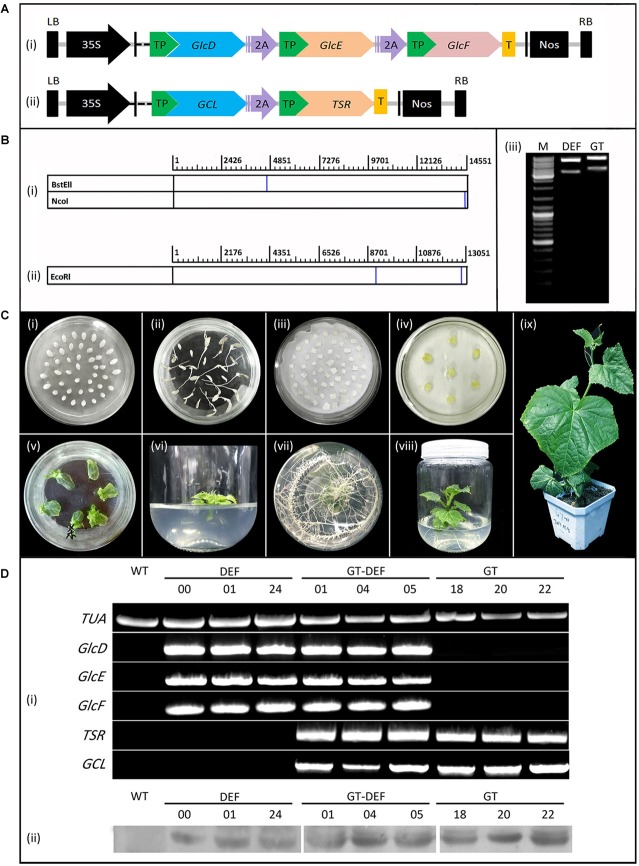
Generation and identification of transgenic plants. **(A)** Schematic diagram of vectors for cucumber transformation. **(i)** DEF expression vector. **(ii)** GT expression vector. 2A: 2A linker peptide. TP, Target peptide; T, The flag protein tag. **(B)** Enzyme cleavage site position and enzyme digestion verification result of expression vectors. **(i)** Enzyme cleavage site position of. DEF vector. **(ii)** Enzyme cleavage site position of. GT vector. **(iii)** Enzyme digestion verification result of DEF and GT vectors. M: 10 Kb marker ladder. **(C)** The construction of cucumber transformation. **(i)** Cucumber seeds, **(ii)** Seed germination, **(iii)** Agrobacterium infection, **(iv)** Elimination of Agrobacterium, **(v)** Bud regeneration, **(vi)** Root induction, **(vii)** Roots, **(viii)** Regeneration of seedling, **(ix)** Seedling. **(D)** Characterization of cucumber transgenic lines by PCR and Western blotting. The upper parts are the results of PCR detection, and the lower part are the results of western blotting. WT, The wild-type cucumber leaves. TUA, The reference gene. Different numbers represent plants from representative transgenic lines.

To achieve co-expression of *GlcD*, *GlcE*, and *GlcF*, the “2A” linker peptide (Ha et al., 2010) was added between *GlcD* and *GlcE*, and between *GlcE* and *GlcF* to construct a fusion gene. Similarly, the “2A” was added between *GCL* and *TSR*. In order to express the gene in the chloroplast, a SSU leader peptide from *A. thaliana* (Lee et al., 2006) was added to the 5′ end of each gene. The constructed *GCL*-*TSR* and *GlcD*-*GlcE*-*GlcF* fusion genes were separately inserted into the NcoI and BstEII restriction sites of pCamBIA 1305.1, and two polycistronic expression vectors (GT and DEF) driven by the 35S promoter were constructed. Vector structures are shown in Figure 2. The fusion gene fragment in the expression vector was relatively long. Thus, the fusion gene expression vector was first verified by enzyme digestion and then sequenced.

### Genetic Transformation of Cucumber

The expression vector carrying DEF and GT was transferred into *Agrobacterium* C58C1 by electroporation (Wang et al., 2015b), and the Agrobacterium-mediated cotyledon transformation method was used for genetic transformation of cucumber. The DEF and GT transgenic plants were infected with *A. tumefaciens* carrying DEF and GT, respectively, and the GT-DEF transgenic plants were co-infected with a mixed *A. tumefaciens* liquid (DEF:GT = 1:1) (Bi et al., 2017). The genetic transformation method of cucumber was conducted as described previously and improved (Wang et al., 2015a).

Cotyledonary explants were prepared as described previously (Wang et al., 2015b). Seed coats were removed with scalpel and forceps after 6 h immersed into sterile distilled water and were then sterilized for 30 s using 75% alcohol. After 15 min sterilizing in 1% (w/v) sodium hypochlorite and rinsing 5 times with sterile distilled water, the dried seeds were germinated at 28 °C in the dark for 2 day in Murashige-Skoog (MS) medium (Liu et al., 2018). When the regenerated shoots have grown to 2 cm, the proximal end of cotyledon explants were pricked and the cotyledon explants were immediately subjected to *Agrobacterium* infection. *Agrobacterium* was cultured in 50 mL YEB at 28°C until optical density at 600 nm (OD600) of 0.6–0.8 was achieved. The *Agrobacterium* culture was centrifuged and resuspended in MS medium to achieve a final OD600 value of 0.2–0.3. The prepared dissected explants were immersed in the *Agrobacterium* inoculum for 15 min, and excess *Agrobacterium* suspension was removed using sterilized filter paper. Infected explants were finally placed in the shooting medium in a dark chamber for 3 days. Explants were then transferred to a rooting medium to induce the regeneration of shoots. After culturing on the selection medium for 2 weeks, the regenerated shoots were excised, transferred to a root regeneration medium, and grown in a lighted chamber at day/night temperatures of 25°C/18°C for root regeneration (Zhang et al., 2014).

### Expression of Glycolate Catabolic Pathway Enzymes in Cucumber

The total soluble protein of the third true leaf of transgenic cucumber plants were extracted, and the GCL, TSR, GlcD, GlcE, and GlcF protein contents of the transgenic plants were determined by enzyme-linked immunosorbent assay (ELISA) kit (Huada Protein, China) (Dalal et al., 2015). The antibody information of each gene is Supplementary Table S1.

### Plant Growth Conditions

**FIGURE 3:**
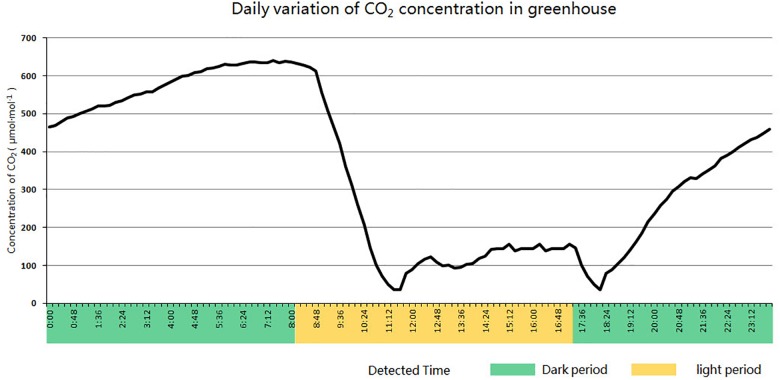
Daily variation curve of CO_2_ concentration in an enclosed solar greenhouse for cucumber cultivation.

After PCR detection and Western blot analysis, transgenic cucumber plants were transplanted from tissue culture flasks into individual 10 cm plastic pots and wrapped in plastic film. Every other day, the membrane was perforated until the transgenic seedlings were adapted to the external environment. Plants were transplanted to larger strip plastic pots (77 cm long, 22 cm wide, and 26 cm deep). Each pot contains 30 l of turfy soil and 30 g of N-P-K compound fertilizer (15-15-15) as a base fertilizer. After transplanting, give the plants plenty of water for the first time, and then water them every 5 to 7 days. When plant grows to the flowering stage, supplement potassium fertilizer for plants with GAP instant soluble fertilizer (14-6-30) Plants were cultivated under short-day conditions (8 h illumination and 16 h darkness) in an enclosed solar greenhouse for low concentration CO_2_ treatment. The daily variation curve of CO_2_ concentration in the greenhouse is shown in Figure 3.

### Gas Exchange and Chlorophyll a Fluorescence Parameters Analysis

To investigate the effect of introduction of glycolate catabolic pathway on photosynthetic capacity of cucumber under low CO_2_ environment, the net photosynthetic rate and photosynthetic daily change of the third true leaves were measured by gas exchange method using the Li-6400 photosynthetic apparatus (LI-COR, China) after transgenic cucumber plants were exposed for 5 weeks to a low CO_2_ environment. The determination conditions were: chamber temperature = 25°C, photon flux density = 1400 mmol m^-2^ s^-1^, CO_2_ concentration = 200 μmol⋅mol^-1^, the gas flow rate = 500 mmol s^-1^, The measurement indicators include: net photosynthetic rate (Pn, μmol⋅m^-2^⋅s^-1^), stomatal conductance (Gs, mol⋅m^-2^⋅s^-1^) and intercellular CO_2_ concentration (Ci, μmol⋅mol^-1^).

The gas exchange parameters were measured from 9:00 am to 11:00 am in sunny days, and the photosynthetic curve was measured from 8:00 am to 5:00 pm. The maximum photosynthetic efficiency of photosystem II (PSII) and the actual photosynthetic efficiency of PSII were determined by chlorophyll fluorescence method using a MINI-PAM chlorophyll fluorescence instrument (WALZ, Germany). The maximum photosynthetic efficiency of PSII (YII) and the actual photosynthetic efficiency of PSII (Fv/Fm) were measured at 7:00 pm.

### Enzyme Activity Assay

To study the effects of the introduction of glycolate catabolic pathway on the photosynthetic metabolism and enzymatic activity in cucumber, four enzyme activities were measured. Carbonic anhydrase (CA), which is responsible for transporting CO_2_ diffused into plant cells to the carboxylation center of RuBisCO (DiMario et al., 2016). RuBisCO, the key enzyme for carbon fixation; RuBisCO activase (RCA), which regulates the activity of RuBisCO enzyme (Bi et al., 2017; Mcfarlane et al., 2018). Glycolate oxidase (GO), the key enzyme in the oxidation pathway of glycolic acid, which affects the rate of photorespiration (Long et al., 1994; Kang et al., 2018). The third true leaves of transgenic cucumber plants were selected for enzyme extraction. Fresh leaves tissue samples were pulverized with liquid nitrogen for CA, RCA, RuBisCO, and GO enzyme extraction.

Carbonic anhydrase was extracted with a Hepes-KOH extract (pH = 8.3, containing 10 mmol⋅L^-1^ of DTT). The activity of CA was measured by a pH method (Rengel, 1995). Added 0.5 mL CA enzyme solution into 4.5 mL of pre-chilled CO_2_-saturated water. Monitoring the pH change of the reaction system with the FE28 pH meter (METTLER TOLEDO, Switzerland). Record the time for the reaction system to drop by one pH unit and the time was recorded as t_1_. The boiled enzyme solution was as a control, and the time was recorded as t_0_. The CA enzyme activity unit (U) is calculated as U = 10 (t_0_/t_1_-1).

RuBisCO was extracted with PBS buffer (pH = 7.4). The activity was measured by ELISA method using the RuBisCO enzyme activity detection ELISA kit (Huada Protein, China). The antibody information was listed in the Supplementary File S1 and Supplementary Table S1.

RuBisCO activase was extracted with RCA enzyme continuous cycle colorimetric quantitative detection kit (GMS16016) and measured by spectrophotometry using the UV-1800 double beam ultraviolet spectrophotometer (SHIMADZU, Japan) (Pan et al., 2014). The detection wavelength is 345 nm. Determine the background of the sample according to the kit instructions and calculate the RCA activity.

Glycolate oxidase was extracted with Tris–HCl buffer (pH = 7.8, containing 0.01% Triton X-100, 5 mmol⋅L^-1^ DTT) and the activity was measured by spectrophotometry using the UV-1800 double beam ultraviolet spectrophotometer (SHIMADZU, Japan) (Booker et al., 1997). Added 0.3 mL GO enzyme solution into 4.5 mL reaction solution (0.009% Triton X-100, 50 mmol⋅L^-1^ Tris–HCl, 3.3 mmol⋅L^-1^ phenylhydrazine HCl) and 5 mmol⋅L^-1^ glycolic acid (neutralized to pH 7.0 with KOH) to start the reaction (the same volume of water was used instead of glycolic acid as the control). Glycolate oxidase activity was determined by following the formation of glyoxylate phenylhydrazone at 324 nm for 5 min.

### Statistical Analysis

The data are presented as the means ± one standard deviation (SD) of three replicates. The statistical analyses were analyzed with one-way ANOVA and performed by the Data Processing System (DPS) (Park and Tuell, 2010). The *Post hoc* Tukey test was applied to analysis significant differences between different treatments.

## Results

### Establishment of Glycolate Catabolic Pathway in Cucumber

To achieve co-expression of multigene, two polycistronic expression vectors were constructed using “2A,” one of which encodes the three subunits GlcD, GlcE, and GlcF of GlcDH (Figure 2Ai). The other encodes GCL and TSR (Figure 2Bii). Each vector contains the SSU target peptide for mediating target genes expression in chloroplasts. The enzyme digestion verification (Figure 2B) and sequencing results (Supplementary Table S2) showed that the expression vector was successfully constructed. The *Agrobacterium*-mediated method was used for the generation of cucumber genetic transformation (Figure 2C). Plants were transformed either by the GT and DEF constructs alone or co-transformed with GT and DEF constructs.

To detect whether the transgenic plants were successfully constructed, cucumber transformation plants were selected by PCR and Western blot analyses (Supplementary Figure S1). Seven GT plants (11, 13, 14, 15, 18, 20, and 22), eight DEF plants (00, 04, 05, 10, 13, 20, 21, and 24), and four GT-DEF plants (01, 04, 05, and 08) were positively detected from the transformants (Figure 3) with the transformation rates of 0.28%, 0.32%, and 0.16% (Figure 2D).

**FIGURE 4:**
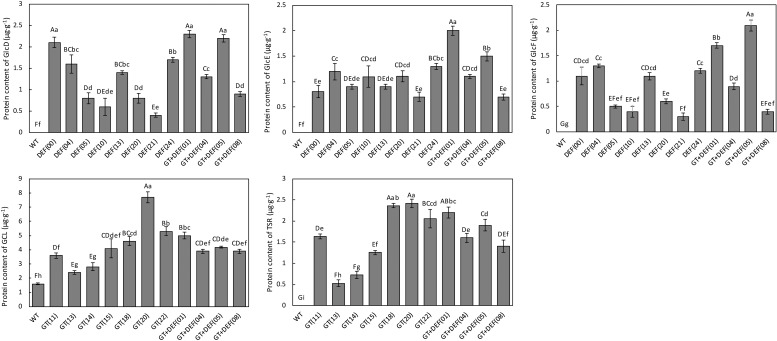
ELISA analysis of target gene coding protein content in different transgenic lines.

To detect the expression of the gene-encoded protein in transgenic plants, the contents of GCL, TSR, GlcD, GlcE, and GlcF were determined by the ELISA assay. All the corresponding proteins were detected in the GT, DEF, and GT-DEF transgenic plants, whereas only the GCL was detected in WT plants. The proteins encoded by other genes were not detected (Figure 4).

### The Glycolate Catabolic Pathway Promoted Cucumber Plant Growth in a Low-CO_2_ Environment

**FIGURE 5:**
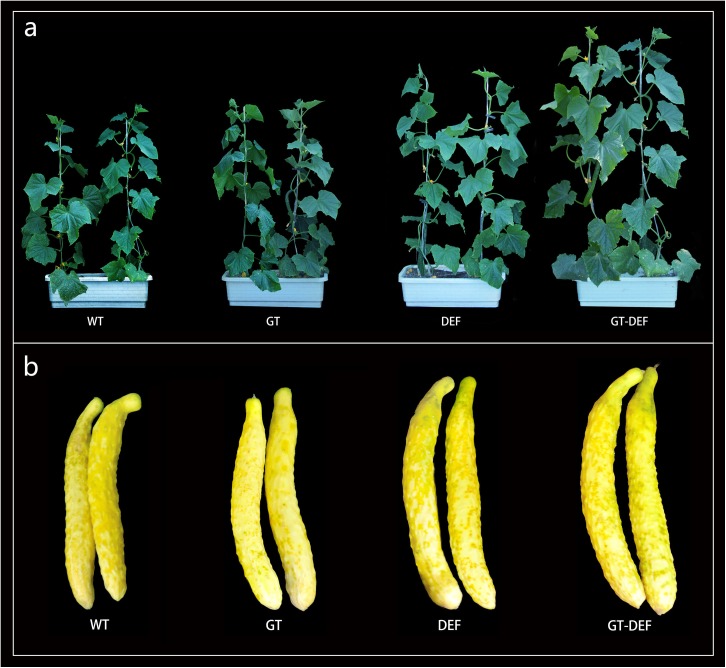
Typical Photographs of cucumber growth in low CO_2_ concentrations. **(a)** Morphological comparison of different transgenic cucumber lines after 6 weeks-growth. **(b)** Comparison of single melon morphology of different transgenic cucumber lines.

**FIGURE 6:**
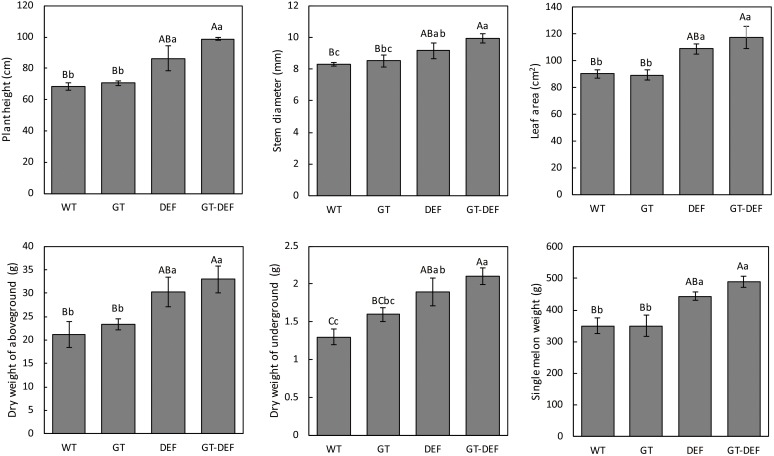
Comparison of the growth parameters between overexpression lines and the control plants after 6 weeks-growth in a low CO_2_ environment. Values represent the means ± SD (*n* = 3) of three plants per line. Capital letters in each figure represent extremely significant differences among samples by Student’s *t*-test (*P* < 0.01) and small letters represent significant differences (*P* < 0.05). Labels in the figures and tables below are the same.

To study the effects of the introduction of glycolate catabolic pathway on the growth of cucumber in low concentration CO_2_ environment, the transgenic plants were transferred to an enclosed greenhouse for low CO_2_ treatment. The plant height, stem diameter, leaf area, single melon weight, and dry weight of the transgenic plants were determined. Plant height, stem diameter, leaf area, dry weight, and single melon weight of DET and GT-DEF transgenic plants were significantly higher than those of WT, and GT-DEF showed better growth characteristics than DEF transgenic plants (Figure 5). Most of the growth indicators of GT transgenic plants were not significantly different from the control (Figure 6).

### The Glycolate Catabolic Pathway Increased the Photosynthetic Capacity of Cucumber Growth in Low CO_2_ Environment

**FIGURE 7:**
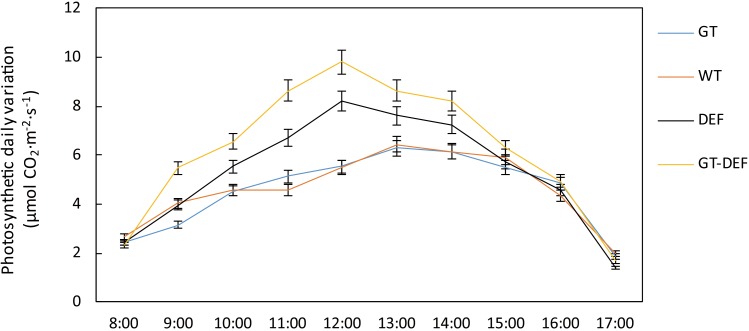
Photosynthetic diurnal variation curve of transgenic plants and wild type control plants after 5 weeks-growth in a low CO_2_ environment.

In order to investigate the effects of the introduction of the glycolate catabolic pathway on the photosynthetic capacity of cucumber in a low CO_2_ environment, we determined the gas exchange parameter of transgenic cucumber plants. The net photosynthetic rates of DEF and GT-DEF plants were 15.0% and 33.4% higher than that of the WT control, respectively, whereas the net photosynthetic rate of GT plants was not significantly different from the control. The photosynthetic diurnal curve also showed a similar trend (Figure 7). The stomatal conductance of DEF and GT-DEF plants were 15.8% and 23.7% higher than WT control, but the deviations of DEF from WT control were not significant. Similarly, we analyzed the intercellular CO_2_ concentrations of different transgenic plants. The results showed that the intercellular CO_2_ concentrations in the leaves of DEF and GT-DEF transgenic plants was significantly higher than that of the wild type control. Then, we measured the maximum photosynthetic efficiency and actual photosynthetic efficiency of PSII by using a chlorophyll fluorescence spectrometer. The actual photosynthetic efficiency of PSII (YII) in DEF and GT-DEF plants was significantly higher than in WT controls, whereas there was no significant difference between GT plants and WT controls. The maximum photosynthetic efficiency (Fv/Fm) of each transgenic plant was not significantly different from the WT control (Table 1).

**Table 1:** Gas exchange parameters and chlorophyll fluorescence of wild-type and transgenic lines.

	WT	GT	DEF	GT-DEF
Pn (μmol⋅m^-2^⋅s^-1^)	5.67 (±0.32)	5.55 (±0.55)	6.52 (±0.60)	7.57 (±0.34)^∗∗^
Gs (mol⋅m^-2^⋅s^-1^)	0.38 (±0.02)	0.38 (±0.03)	0.44 (±0.02)	0.47 (±0.02)^∗^
Ci (μmol⋅mol^-1^)	146.32 (±3.30)	149.64 (±0.84)	155.97 (±1.24)^∗^	157.77 (±3.76)^∗^
Fv/Fm	0.69 (±0.012)	0.70 (±0.011)	0.71 (±0.010)^∗^	0.71 (±0.005)^∗^
YII	0.12 (±0.008)	0.13 (±0.016)	0.18 (±0.007)^∗∗^	0.21 (±0.005)^∗∗^

*Values represent the means ± SD (n = 3) of three plants per line. ^∗^P < 0.05; ^∗∗^P < 0.01 according to Student’s t-test.*

### The Glycolate Catabolic Pathway Increased the Activity of Photosynthetic Carbon- Fixation Enzymes and Reduced the Activity of Photorespiration Enzymes in Transgenic Plants

**FIGURE 8:**
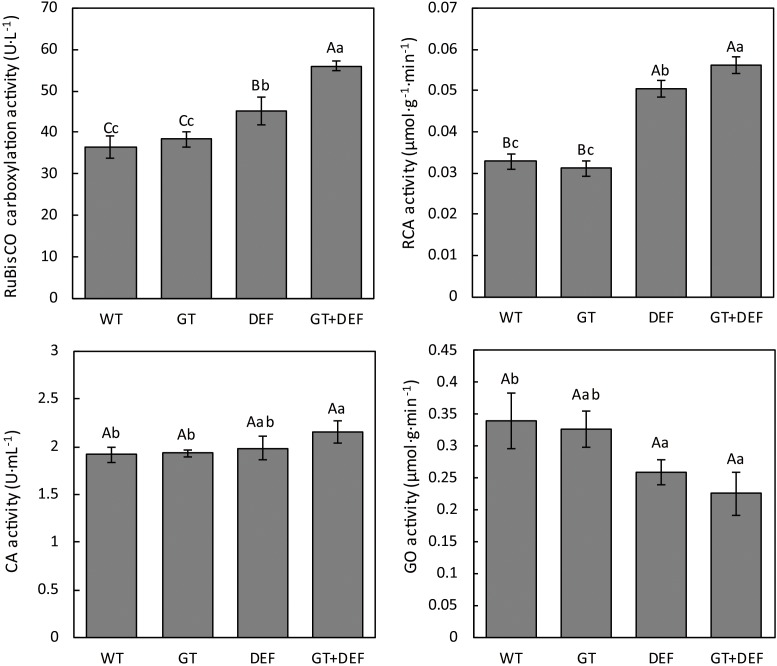
Comparison of photosynthetic enzyme activities between transgenic plants and wild type control plants after 5 weeks-growth in a low CO_2_ environment.

To further investigate the intrinsic mechanism of glycolate catabolic pathway, which promotes cucumber photosynthesis and growth, the activities of RuBisCO, CA, RCA, and GO in transgenic plants were determined. RuBisCO, which catalyzes CO_2_ fixation in carbon assimilation, and RCA enzyme, which activates RuBisCO, showed higher enzymatic activity in DEF and GT-DEF plants than in WT controls. CA is mainly present in the chloroplast stroma of mesophyll cells in C3 plants. This localization is related to the diffusion of CO_2_ across the chloroplast membrane and the rapid dehydration of H_2_CO_3_ to free CO_2_ to maintain sufficient CO_2_ concentration around RuBisCO (DiMario et al., 2016). The CA activity results showed that only GT-DEF transgenic plants exhibited higher activity than WT controls, whereas there was no significant difference in DEF, GT, and WT controls. Glycolate oxidase (GO) is a key enzyme in the oxidation pathway of glycolic acid, and its activity affects the rate of photorespiration (Long et al., 1994; Kang et al., 2018). The results of GO assay showed that the GO activity in DEF and GT-DEF transgenic plants was significantly lower than in WT controls, whereas the GO activity of GT transgenic plants was not significantly different from the control (Figure 8).

## Discussion

There are two types of photosynthetic cells, namely mesophyll and leaf sheath cells, in the leaves of C4 plants. Thin-walled mesophyll cells surround the sheath cells, thereby forming a Kranz structure that is distinct from C3 plants (P’yankov et al., 1997). The receptor for CO_2_ in the C4 pathway is PEP in the cytoplasm of mesophyll. CO_2_ is immobilized by PEP carboxylase to form C4 acid (Malic acid or Aspartic acid). In the cytoplasm of mesophyll, PEP carboxylase catalyzes the reaction of phosphoenolpyruvate with CO_2_ to form C4 acid. The C4 acid is then transported to the leaf sheath cells for decarboxylation, and the released CO_2_ is re-fixed by RuBisCO. The Kranz structure is like a CO_2_ pump that transports CO_2_ around RuBisCO (Leegood, 2002). Compared with C3 plants, the CO_2_ around the C4 plant RuBisCO is concentrated, which not only improved the CO_2_ assimilation efficiency but also inhibited the competition of O_2_ for RuBisCO. Thus, C4 plants exhibited higher photosynthetic rate and lower photorespiration than C3 plants (Jeanneau et al., 2002). Such difference is particularly evident under low CO_2_ concentration conditions. According to reports, C3 plant photorespiration consumption can reduce photosynthetic efficiency by nearly 40% (Matsuoka et al., 2001).

High efficiency breeding by introducing C4 plant-related enzymes into C3 plants, knocking out photorespiration metabolic genes, and chemically inhibiting photorespiration has become a research hotspot to improve the yield of C3 crops (Ku et al., 1999; Jiao et al., 2002; Fukayama et al., 2003). However, the introduction of the C4 pathway-related enzyme gene or inhibition of photorespiration cannot improve the photosynthetic efficiency of C3 plants and even affects the normal growth of plants (Ku et al., 1999; Takeuchi et al., 2000; Agarie et al., 2002; Fukayama et al., 2003). The reason for this may be multifaceted. First, although the C4 pathway-related enzyme genes can be highly expressed in C3 plants, the expression products may not be activated. Second, C4 photosynthetic related genes are specifically expressed at the cellular level. There is no Kranz structure in C3 plants. Thus, the expression of C4 photosynthetic genes introduced into C3 plants does not have this specific expression characteristic in C3 photosynthetic cells. Moreover, the C4 pathway involves the coordinated expression of multiple genes. Thus, single expression of C4 photosynthetic gene may not alter the photosynthesis mechanism of C3 plants. During greenhouse vegetable production in winter, the greenhouse is generally not ventilated or only ventilated at a high temperature (noon time) to ensure the temperature of the greenhouse and avoid heat loss caused by the exchange of greenhouse and external airflow during greenhouse ventilation (Kimball and Mitchell, 1979). Vegetable crops are grown in a low CO_2_ environment in the morning and afternoon, and the greenhouse light and heat resources are not fully utilized (Wei et al., 2002). In addition, due to the large leaf area index and the strong photosynthesis of cucumber, the photosynthetic reduction and photorespiration enhancement of cucumber were more obvious in a low CO_2_ environment (Zhang et al., 1997). Researchers introduced the *E. coli* glycolate catabolic pathway into C3 plants, which significantly increased photosynthetic and biological yields of C3 plants (Kebeish et al., 2007; Nölke et al., 2014; Dalal et al., 2015). Inspired by this, this study introduced the glycolate catabolic pathway into cucumber by expressing the genes related to glycolate catabolic pathway of *E. coli* in cucumber chloroplasts. Transgenic plants showed significant photosynthesis and growth advantages over WT controls. By measuring the activity of enzymes related to carbon metabolism in transgenic plants, we found that the introduction of glycolate catabolic pathway significantly increased the activity of RuBisCO and RCA and reduced the activity of GO but had little effect on carbonic anhydrase activity. These results indicated that the introduction of exogenous glycolate catabolic pathway could not improve the ability of cucumber to absorb low concentrations of CO_2_ from environment but enhanced the CO_2_ fixation of cucumber by increasing the activity of RuBisCO and reducing photorespiration. In addition, the introduction of glycolate catabolic pathway promotes the decomposition of glycolic acid in photorespiration products and reduces the energy and carbon consumption caused by the metabolism of glycolic acid. The glycolic acid decomposition product CO_2_ promotes RuBisCO activity. At the same time, it provides carbon sources for cucumber carbon assimilation. The photorespiration substrate-glycolic acid is consumed by GDH. Thus, the GO activity is reduced, thereby resulting in a decrease in photorespiration, which ultimately improves the net photosynthetic rate of cucumber in a low concentration CO_2_ environment, thereby improving cucumber growth. Among the three transgenic lines, GT transgenic plants did not show significant differences with the control in most of the photosynthesis, morphological, and enzymatic indicators, whereas DEF and GT-DEF transgenic plants showed significant advantages compared with the control. It is proved that the introduction of GDH enzyme in the glycolate catabolic pathway plays an important role in promoting plant growth under low CO_2_ conditions. Cucumber has GCL enzyme. Thus, the introduction of GDH enzyme into cucumber can realize the metabolism process of glycolic acid to CO_2_. The reaction provides CO_2_ to RuBisCO, which increases the carboxylation activity of RuBisCO and promotes the Calvin cycle reaction. The GT-DEF transgenic plants showed higher photosynthesis and growth than the DEF plants, which proved that the exogenous glycolate catabolic pathway played an active role in the plants. The results of enzyme activity assay and photosynthetic fluorescence confirmed the cause of this effect. The introduction of the glycolate catabolic pathway consumes the photorespiration substrate glycolic acid, and the CO_2_ produced increases the carboxylation activity of RuBisCO. The 3-PGA produced provides a carbon source for the Calvin cycle to promote carbon assimilation. The dual effects of photosynthetic enhancement and photorespiration attenuate improved the growth of cucumber in low CO_2_ environments.

## Author Contributions

Z-fC, SS, and G-mX conceived and designed the research. Z-fC, T-lZ, and DZ performed the experiments. Z-fC and H-mN carried out the genes expression analysis. Z-fC, S-wZ, T-lZ, and H-mN analyzed the data. All authors contributed to writing this manuscript and approved its final version.

## Conflict of Interest Statement

The authors declare that the research was conducted in the absence of any commercial or financial relationships that could be construed as a potential conflict of interest.
