# Supplementary material for: Introduction of Exogenous Glycolate Catabolic Pathway Can Strongly Enhances Photosynthesis and Biomass Yield of Cucumber Grown in a Low-CO2 Environment
Source: Front Plant Sci. 2019 May 29;10:702. doi: 10.3389/fpls.2019.00702 (PMC6549358; doi:10.3389/fpls.2019.00702)
Supplement: Supplementary file 1 [file Table_1.DOCX]

**Introduction of** **exogenous glycolate catabolic pathway can strongly enhances photosynthesis and biomass yield of cucumber grown in a low-CO_2_ environment**

***Zhi-feng Chen^1,2^, Hong-mei Nie^1,2^, Shao-wen Zheng^1,2^, Tian-li Zhang^1^, Dan-zhou^1^, Guo-ming Xing^1,2*^, Sheng Sun^1,2*^***

***1:*** *College of Horticulture, Shanxi Agricultural University, Taigu, Shanxi,030800, China*

***2:*** *Collaborative Innovation Center for improving the Quality and efficiency of Greenhouse Vegetable in Shanxi Province, Taigu, Shanxi,030800, China*

***Running title****:* *Improve photosynthesis and biomass yield of cucumber under low-CO_2_ environment*

****Corresponding author:***

*Dr. Guo-ming Xing*

Director and Professor

The Collaborative Innovation Center for improving the Quality and efficiency of Greenhouse Vegetable in Shanxi Province,

College of Horticulture, Shanxi Agricultural University.

No. 1, Xingnong Street, Mingxian South Road, Taigu, Shanxi, People’s Republic of China,

Email address: xingguoming@163.com(G.M. X)

**Table S1 Antigen sequences for ELISA**

| Protein | Antigen sequences |
| --- | --- |
| GCL | QAPRARLHKEDFQAV |
| TSR | ETARQVTEASD |
| GlcD | RLAQDEAERVRFW |
| GlcE | AGPRRPWSGSVR |
| GlcF | ARQLRDNKMNALE |

**Table S2 Primer sequences in this study**

| Genes | Sense primer | Anti-sense primer | |
| --- | --- | --- | --- |
| *TUA* | ATGAGAGAGTGCATCTCAATT | | TTAGTAGTCCTCTCCTTCATCAT |
| *GCL* | ATGGCTAAGATGAGGGCTG | | TCATTCGTAGTGCATAAAACAA |
| *TSR* | ATGGCTAAGATGAGGGCTG | | TCATTCGTAGTGCATAAAACAA |
| *GlcD* | ATGAGTATTTTGTACGAGGAGA  GAC | | TCAGAACCTCTCCAATTCTGG |
| *GlcE* | ATGCTCAGAGAGTGCGATTAC | | TCAAAGTTCAGCGTACATTCT |
| *GlcF* | ATGCAGACCCAACTTACTGA | | TCACTCTTTTTCAAGAGCTTG |

**Figure S1 The picture of the whole membranes of WB**

**
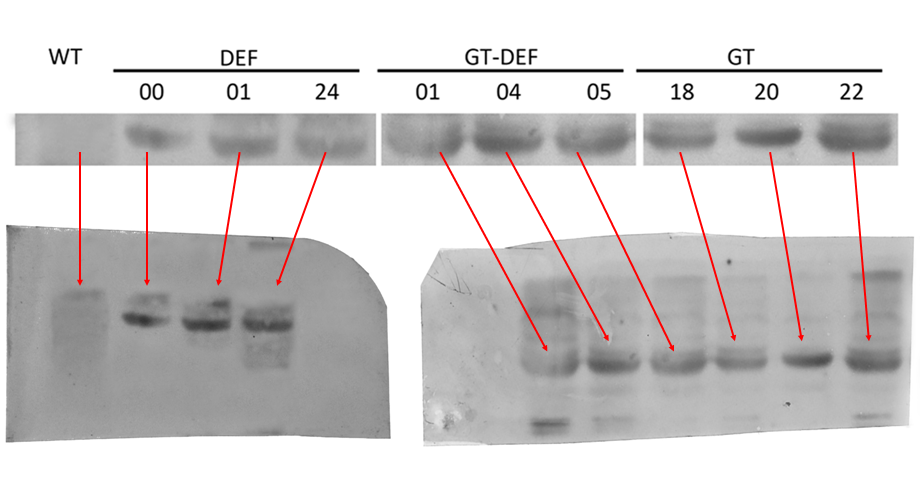
**
